# Risk of sound-induced hearing loss from exposure to video gaming or esports: a systematic scoping review

**DOI:** 10.1136/bmjph-2023-000253

**Published:** 2024-01-04

**Authors:** Lauren K Dillard, Peter Mulas, Carolina Der, Xinxing Fu, Shelly Chadha

**Affiliations:** 1 Department of Otolaryngology-Head & Neck Surgery, Medical University of South Carolina, Charleston, South Carolina, USA; 2 Department of Noncommunicable Diseases, World Health Organization, Geneva, Switzerland; 3 Beijing Institute of Otolaryngology, Capital Medical University, Beijing, China; 4 Ear Science Institute Australia, Subiaco, Perth, Australia

**Keywords:** Public Health, Preventive Medicine, Public Health Practice, Primary Prevention, education

## Abstract

**ABSTRACT:**

**Background:**

There is little information on whether video gaming might be a modifiable risk factor for hearing loss and/or tinnitus, despite the plausibility of these relationships given that video games are often played at high-intensity sound levels and for long periods of time.

**Objective:**

To synthesise current evidence related to relationships between gaming and the potential risk of hearing loss and/or tinnitus.

**Design:**

Systematic scoping review

**Data sources:**

We searched three databases (PubMed, Scopus, Ovid MEDLINE) in January 2023 for peer-reviewed articles, and searched grey literature sources, from inception to 2023.

**Eligibility criteria:**

Observational, mixed-methods, trials, or case studies published in (or that could be translated into) English, Spanish or Chinese were eligible for inclusion. Studies were included if they evaluated relationships of gaming with hearing loss and/or tinnitus.

**Data extraction and synthesis:**

Two reviewers extracted and verified study data, which are synthesised in tables and in the text.

**Results:**

Fourteen peer-reviewed studies were included, 11 of which were cohort studies and 3 of which were non-cohort observational studies. Across studies, the prevalence of gaming ranged from 20% to 78%. In general, the average measured sound levels of video games nearly exceeded, or exceeded, permissible sound exposure limits, and on average, individuals played video games for approximately 3 hours per week. Among the five peer-reviewed studies that evaluated associations or correlations of gaming with hearing loss or tinnitus, four reported significant associations or correlations with gaming and hearing loss or tinnitus.

**Conclusions:**

The limited available evidence suggests that gaming may be a common source of unsafe listening, which could place many individuals worldwide at risk of permanent hearing loss and/or tinnitus. Additional research on these relationships is needed along with steps to promote safe listening among gamers.

**WHAT IS ALREADY KNOWN ON THIS TOPIC**

**WHAT THIS STUDY ADDS**

- Taken together, the evidence collated in this scoping review suggests that gaming is likely a source of unsafe listening and thus may put many gamers worldwide at risk of permanent sound-induced hearing loss and/or tinnitus.

**HOW THIS STUDY MIGHT AFFECT RESEARCH, PRACTICE OR POLICY**

- Study findings suggest that there is a need to prioritise interventions, such as initiatives focused on education and awareness of the potential risks of gaming on the auditory system, which can help to promote safe listening among gamers worldwide.

## Introduction

 The World Health Organisation (WHO) recognises voluntary recreational exposure to high-intensity sounds, known as unsafe listening, as an important modifiable risk factor of hearing loss.^1^,^3^ The burden of unsafe listening is high and can occur via exposure to several sources of high-intensity sounds, such as personal listening devices and entertainment venues.^2^,^4^ Another potential source of unsafe listening that has received less attention is video gaming, including electronic sports (known as esports).^3^ Gaming may be a source of unsafe listening because individuals often play video games at high-intensity sound levels and for several hours at a time. Furthermore, gaming is one of the most popular leisure activities worldwide, with an online source estimating there were over 3 billion gamers worldwide in 2022.^5^ Identifying common sources of unsafe listening is important because it is possible to reduce unsafe listening practices through known public health practices and by developing and implementing global policies focused on promotion of safe listening practices.^6^

Research suggests that recurring, or even a single, exposure to high-intensity sounds, could permanently damage the auditory system, commonly referred to as sound-induced or noise-induced hearing loss.^7^,^9^ Early signs of sound-induced hearing loss include (1) transient or permanent tinnitus, which can be defined as a perceived sensation, often ringing or buzzing, in the absence of an external stimulus, (2) hyperacusis, which can be defined as enhanced sensitivity to sound, and/or (3) difficulties understanding speech, particularly when background noise is present.^1 10^ Exposure to high-intensity sounds has been tied to 'hidden hearing loss,’ which exists without measurable permanent changes to audiometric thresholds but can present as the symptoms described above.^11 12^ It has been hypothesised that hidden hearing loss is common among young people given that many young people engage in unsafe listening practices.^4 12^ Importantly, exposure to high-intensity sounds among young people might also make them more vulnerable to developing age-related hearing loss later in life.^13 14^ It is important to note that permanent hearing loss and/or tinnitus caused by high-intensity sounds can be prevented. Identifying modifiable risk factors of hearing loss and/or tinnitus, such as unsafe listening, can promote its prevention, thus reducing the burden of these conditions on individuals and society.

There are several ways in which unsafe listening may occur when playing video games. First, individuals can play video games at home through a gaming console, personal computer (PC) or on a smart device (eg, smart phone or tablet). Second, video games can be played over local area networks in gaming centres or PC rooms, which are locations that offer computers or gaming consoles primarily for the purpose of playing multiplayer games. Headphones are frequently used, and individuals may increase the volume to higher levels than their preferred listening levels to overcome the background noise that is common in these settings. While gaming centres and PC rooms exist worldwide, the vast majority are in China and other Asian countries. Third, electronic sports, termed esports, involve competitive, organised gaming and are rapidly growing in popularity. Some esports players are professional gamers, and therefore may practice for many hours a day, often using headphones, and compete regularly in online or in-person competitions or tournaments. Esports competitions or tournaments can be available to spectators either online or in-person at esports arenas. These esports arenas livestream virtual esports events, and some also host esports competitors in person during which spectators watch the competitions. Therefore, both esports gamers and spectators may be at risk for unsafe listening.

Permissible levels of recreational sound exposure can be estimated from equivalent occupational noise exposure limits, which are defined as an average sound intensity (eg, 80 decibels (dB)) over a period of time—for example, a 40-hour working week).^7 15^ Permissible noise exposure limits vary slightly by region or by regulatory agencies or organisations and the target population’s age.^7 16 17^ Here, we focus on noise exposure limits published by the International Telecommunication Union (ITU) in collaboration with WHO.^7^ Sound intensity (dB) is measured on a logarithmic scale and there is a time–intensity trade-off, known as an exchange rate, for permissible levels and duration of exposure, and therefore, permissible levels of noise exposure change drastically by sound level. For example, based on a permissible noise exposure level of 80 dB for 40 hours a week with a 3 dB exchange rate, the permissible exposure time of an 83 dB sound is 20 hours, of an 86 dB sound is 10 hours, of a 92 dB sound is 2.5 hours and of a 98 dB sound is 38 min per week.^7 15 18^ For children, permissible noise exposure levels are lower and can be based on a permissible noise exposure level of 75 dB for 40 hours a week. Under this definition, children can safely listen to sound at an 83 dB sound for approximately 6.5 hours, an 86 dB sound for approximately 3.25 hours, a 92 dB sound for 45 min, and a 98 dB sound for only 12 min per week.^7^

Using an average sound intensity to define permissible levels of sound exposure is common; however, an average does not define whether impulse sounds, which can be defined as sound consisting of bursts lasting less than one second and with peak levels at least 15 dB higher than the background sound, are included in the average.^19^ In occupational settings, impulse noise may have negative effects on hearing, beyond what can be explained by exposure to non-impulse noise, and may lead to acute acoustical trauma.^19 20^ In general, the audio of video games comprises of (1) sounds of similar intensities, and (2) often (particularly in shooter games), impulse sounds. Permissible exposure limits for impulse sounds vary but are approximately 100 dB for children and 130–140 dB for adults.^17 19^

Taken together, it is plausible that gaming may be a source of unsafe listening. Yet, it is not known whether gaming may pose a risk to auditory function. Efforts towards prevention of global hearing loss would benefit from identifying sources of unsafe listening, which may include gaming. Therefore, the objective of this systematic scoping review is to synthesise current evidence related to relationships between gaming and the potential risk of hearing loss and/or tinnitus.

## Methods

This systematic scoping review was conducted under the Preferred Reporting Items for Systematic Reviews and Meta-Analyses guidelines for scoping reviews (PRISMA-ScR).^21^ The protocol was registered with Open Science Framework (registration: https://doi.org/10.17605/OSF.IO/T397A). Given the nature of this review, a critical appraisal of individual sources of evidence was not conducted.

### Eligibility criteria

Peer-reviewed articles or grey literature published in English, Spanish or Chinese (or that could be translated into those languages) that were observational, mixed-methods, trials, or case studies were eligible for inclusion. No eligibility restrictions were placed on year of publication. Studies were included if they evaluated relationships between video games or esports and hearing loss and/or tinnitus or mentioned gaming as a risk factor for hearing loss and/or tinnitus.

### Information sources and search

The electronic databases PubMed, Scopus and Ovid MEDLINE were searched on 17 January 2023. Pilot searches confirmed the sensitivity and specificity of search terms. Search strings are shown in online supplemental file 1. Search terms were translated into Spanish and Chinese, and articles were hand-searched in Google Scholar and SinoMed, respectively. Reference lists and citations of all included studies were searched to identify additional relevant articles.

Grey literature sources, including white papers, newsletters, reports or proceedings, were identified using the keywords (or derivations of) ‘hearing loss,’ ‘noise induced,’ and ‘video games’ or ‘esports.’ Additionally, Google Scholar was searched to detect other grey literature sources, such as dissertations, theses, published abstracts or conference papers.

### Selection of sources of evidence

Titles and abstracts of peer-reviewed articles were screened by a single reviewer. Full-text articles in English were screened by two reviewers, and differences were reconciled by discussion of the articles. Full-text articles in Spanish or Chinese were screened by a single reviewer.

### Data charting process and data items

Data collection tables were developed and piloted to extract data from several relevant articles prior to the conduct of this review. For studies published in English, a single reviewer extracted study data, and data were verified by a second reviewer. For studies published in Spanish or Chinese, one reviewer extracted study data. Data collection tables included details on (a) meta study information (eg, author, year, journal), (b) study and sample characteristics (eg, study design, participant age, location), and (c) results related to gaming and hearing loss and/or tinnitus. Age was categorised based on the age categories defined in the included studies, as follows: children: <19 years, adults: ≥19 years, or adults and children. The charted data were synthesised in the tables presented in the Results section.

### Patient and public involvement statement

Patients or the public were not involved in the design, conduct or reporting of this systematic scoping review.

## Results

The results section is organised as follows. First, we present information from peer-reviewed articles focusing on relationships between gaming and hearing and/or tinnitus, which comprise the main findings from this review. Second, we present information from peer-reviewed articles and grey literature sources that mention, but do not focus on, relationships between gaming and hearing and/or tinnitus. Third, we present additional information from grey literature sources.

**Figure 1:**
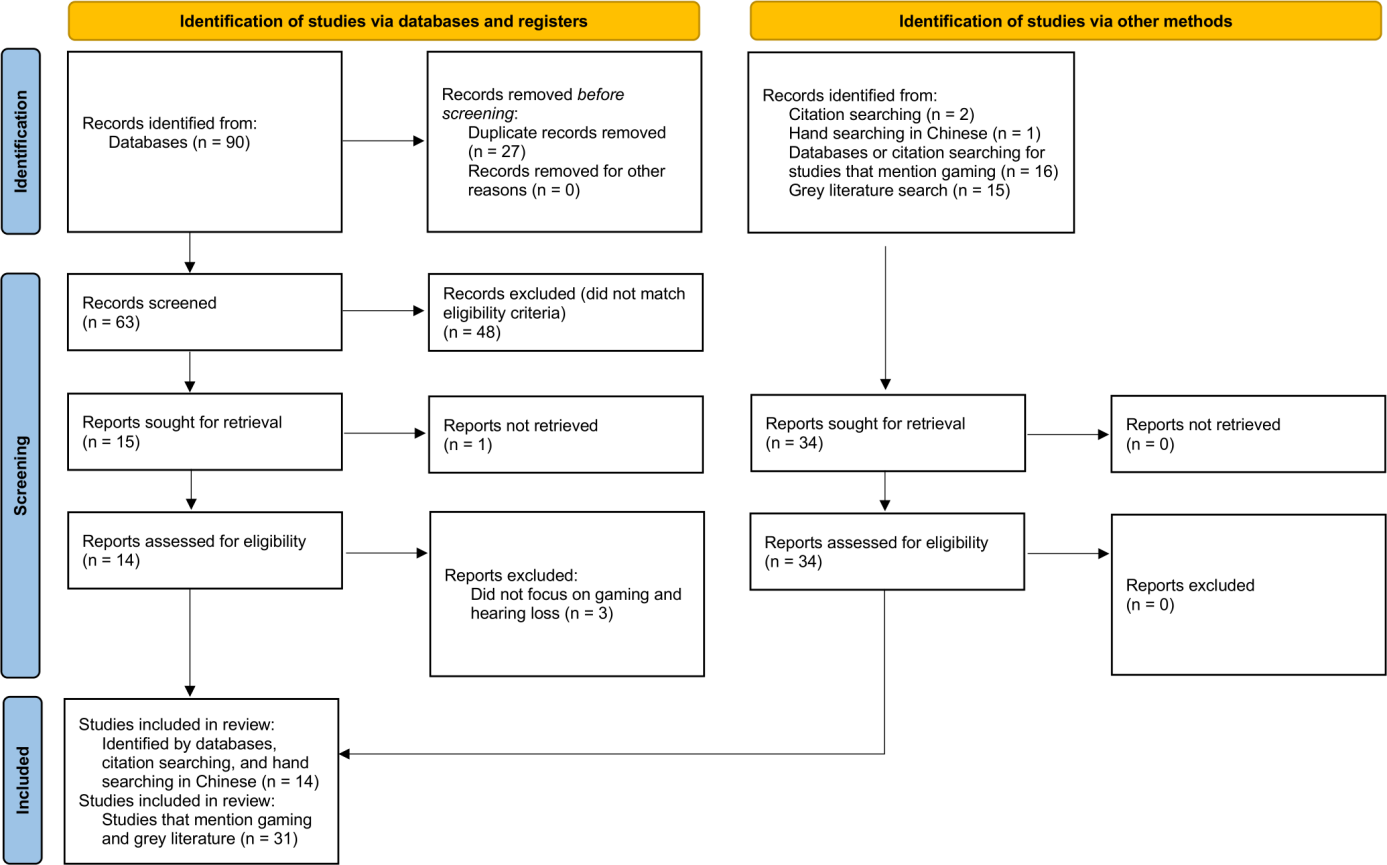
Flow diagram^22^ summarising the article screening process.

A total of 63 non-duplicate peer-reviewed citations were identified that focused on relationships between gaming and hearing and/ or tinnitus. After the final review, 14 peer-reviewed articles were included in this study. The study selection process is shown in figure 1.^22^ Studies were from nine countries, corresponding to representation from American (n = 2), European (n = 4), South-East Asian (n = 6) and Western Pacific (n = 2) regions. Twelve studies were published in English, one was published in Korean and translated to English for review, and one was published in Chinese.

### Cohort studies

Details of the 11 cohort studies that evaluate relationships between gaming and hearing loss and/or tinnitus are given in table 1.^23^,^33^ Ten cohort studies were cross-sectional, and one was longitudinal. Six studies were conducted in children (<19 years),^25^,^30^ three were conducted in adults (≥19 years),^24 32 33^ and two were conducted in adults and children.^23 31^ Six studies evaluated relationships of hearing with computer or video games,^23^,^2629 33^ four studies evaluated relationships of hearing with use of gaming centres or PC rooms^27 28 30 31^ and one study evaluated relationships of hearing with games on mobile devices.^32^

**Table 1:** Pertinent study characteristics from cohort studies that evaluated relationships of gaming and hearing or tinnitus.

First author (year)country	Purpose	Study design	nAge group or mean (range) age	Type and definition of gaming	Definition, hearing measure	Sound level and duration of gaming	Prevalence, gaming	Primary study findings
Beach *et al* ^23^(2016) Australia	Describe use of hearing protection devices in leisure activities.	Cohort (cross-sectional)	8144>15 years	Use of electronic games	Self-report.Severity of hearing loss	--	--	Gaming was associated with poorer self-reported hearing.Use of hearing protection is uncommon (<5%) among gamers.
Bhatt *et al* ^24^(2017) USA	Identify sources of sound exposure and hearing protection use.	Cohort (cross-sectional)	36 697(extrapolated to 240 million)*47 years	At least 10 exposures to video or computer games in past year.Classified as ‘loud’ or ‘very loud.’	--	--	--	Over 10 million individuals in the USA may be exposed to ‘loud’ or ‘very loud’ sound from games.*
Dehnert *et al*^25^ †(2015) Germany	Investigate total leisure sound exposure in adolescents and its association with hearing. Determine whether sound exposure exceeds yearly acceptable exposure.‡	Cohort (cross-sectional)	214315.4 (13–19) years	Self-reported play of video games via headphones in last year	Audiometric high- and low-frequency hearing loss‡	Duration (median): 3.1 hours/week	35.4%	Sound exposure from gaming did not exceed acceptable yearly exposure.§Boys (vs girls) have higher prevalence of gaming and spend more time gaming.
Dreher *et al*^26^ †(2018) Germany	Investigate leisure sound exposure and associated sociodemographic determinants.	Cohort (longitudinal with three waves)	21489–14 years	Self-reported play of video games via headphones in last year	Self-report	Duration (mean):W1: 3.1 hours/week,W2: 3.5 hours/week,W3: 3 hours/week	W1: 26.1%W2: 20.9%W3: 19.7%	Gaming is common. Boys (vs girls) have higher prevalence of gaming.
Rhee *et al*^27^ ¶(2019) South Korea	Evaluate prevalence of hearing loss among adolescents and associated risk factors.	Cohort (cross-sectional)	2979Middle and high school students	Use of gaming centre (in past year)	Audiometric speech-frequency hearing loss: PTA (0.5, 1.0, 2.0 kHz ≥15 dB) in both ears; high-frequency hearing loss: PTA (0.3, 4.0, 6.0, 8.0 kHz ≥15 dB) in both ears	Duration:95% of those who reported visiting gaming centres did so for >1 hour per visit	59.8%	Use of gaming centres was associated with increased odds of bilateral high- frequency hearing loss.There was a positive correlation between hours spent in gaming centres and prevalence of high- frequency hearing loss.
Rhee *et al*^28^ (2020) ¶South Korea	Estimate prevalence of tinnitus among adolescents and associated risk factors.	Cohort (cross-sectional)	1593Middle and high school students	Use of gaming centre (in past year)	Self-report.Severe tinnitus defined as tinnitus that was annoying, bothersome, or caused sleep problems.	--	59.9%	Use of gaming centres was associated with increased odds of severe tinnitus.
Swierniak *et al*^29^ (2020)Poland	Identify sources of sound exposure among children.	Cohort (cross-sectional)	103211–12 years	Video games	High-frequency audiometric notch‡	Duration:Daily (14.4%),4–5 x/week (14.1%),2–3 x/week (30.5%),1 x/week (19.3%),1 x/mo (12.5%), several times/ year (9.2%)	78%	Video gaming is a common source of leisure sound exposure (second to personal listening devices).
Shin *et al*^30^ (2005)South Korea	Evaluate sound exposure among young people going to PC rooms.	Cohort (cross-sectional)	120Middle and high school students	PC rooms	--	Sound level (measured at ear level with sound level metre): minimum >80 dB(A), maximum <90 dB(A).Duration using headphones in PC rooms: 1–2 hours**	--	PC games were commonly played through headphones and at a high-intensity sound level.Males (vs females) were more likely to play PC games and listen through headphones at a higher-intensity sound level.
Wicaksono *et al*^31^ (2018)Indonesia	Evaluate correlation between visiting a gaming centre and hearing thresholds.	Cohort (cross-sectional)	1620.4 (SD 1.03) years	Gaming centre	Audiometric threshold at 4.0 kHz	Sound level (measured with sound level metre) (mean): 84.5 dB(A).Duration (mean): 3 hours/visit, and 5.2 x/month.	--	There was a correlation between the number of gaming centre visits per month with threshold at 4.0 kHz in the right ear only.
Yu *et al*^32^ (2016)South Korea	Measure preferred listening levels of mobile phones considering subway interior noise.	Cohort (cross-sectional)	74Range 20–>60 years	Game on mobile device	--	Sound level (mean): 43.2 dB(A)	--	Preferred listening levels for games on mobile devices in the presence of subway background noise were within acceptable noise exposure limits.
Zhang *et al*^33^ (2019)China	Evaluate factors associated with hearing and auditory symptoms among medical students.	Cohort (cross-sectional)	188220.4±1.3 years	Video games	Audiometric speech-frequency hearing loss: PTA (0.5, 1.0, 2.0, 4.0 kHz≥25 dB) in either ear; high-frequency hearing loss: PTA (3.0, 4.0, 6.0, 8.0 kHz≥25 dB) in either ear	--	29.1%	Gaming was associated with increased odds of aural fullness, but not speech- or high-frequency hearing loss.

* Extrapolated to representative statistics for the national population in the USA by using survey weights and survey statistics accounting for weighted and stratified survey design.

† Studies conducted using data from the same cohort (OHRKAN).

‡ Measured hearing loss but did not evaluate its association with gaming.

§ Defined as E_A,T_ = 4 * T_j_ * 100.1(Leq,j-100). Where E_A,T_ is the noise exposure level expressed in Pascal squared hours (Pa2h), T_j_ is the time spent in an activity j hours per week, and L_eq,j_ is the sound pressure level associated with activity j. Assumptions are that an 8-hour hour working day (220 working days/year) with an acceptable L_Aeq,8h 85 dB(A)_ will result in an acceptable yearly exposure of about 222.6 Pa2h Pa2h. A ratio of the noise exposure level of an activity to the acceptable noise exposure >1 interpreted as exceeding the acceptable yearly exposure. The average sound level was not measured, but rather was estimated from a study conducted in arcade rooms.^35 36^

¶ Studies conducting using data from the same cohort.

** Includes all activities in PC PC rooms, including video games and listening to music.

dB, decibel; dB(A), decibel, A weighted; kHz, kilohertz; PC, personal computerPTA, pure-tone average

Of the studies that measured hearing, it was measured by self-report and audiometry in two and five studies, respectively. One study evaluated the outcome of self-reported tinnitus.^28^

Five studies evaluated associations or correlations between gaming and hearing or tinnitus.^23 27 28 31 33^ The other six studies did not evaluate associations, and rather, described other relevant information, such as the frequency and time spent gaming, sex differences in gaming practices and/or how many individuals may be exposed to high-intensity sounds from gaming.^24^,^2629 30 32^ Two studies focused on the use of hearing protection during activities with high-intensity sounds, including gaming.^23 24^

#### Sound level and duration of gaming

Details on the sound levels and duration of gaming are in table 1. Three studies reported the sound levels of gaming, as measured by a sound level metre or device output.^30^,^32^ In a study that focused on mobile device gaming, the average sound level of the games was 43.2 dB(A).^32^ In studies focused on gaming centres, one study reported the average sound level to be 84.5 dB(A); another study reported the minimum and maximum sound level to be >80 dB(A) and <90 dB(A), respectively.^30 31^ Those two studies focused on gaming centres also reported some details on duration of exposure. One study reported that on average, participants visited gaming centres for 3 hours per visit, and 5.2 times per month, and the other study reported participants used headphones in gaming centres for an average of 1–2 hours per visit.^30 31^

Four additional studies included details on duration of exposure, which were self-reported by participants (table 1). Two studies, conducted as part of the OHRKAN study,^25 26^ reported that participants played video games via headphones (1) for a median of 3.1 hours/week (cross-sectional analysis),^25^ and (2) for a mean of 3.1, 3.5, and 3.0 hours/week (longitudinal analysis) across three study cycles conducted over 5 years.^26^ In another cohort, authors reported that among those who play video games, 14.4% play daily, 14.1% play 4-5 times per week, 30.5% play 2–3 times per week, 19.3% play once a week, and the remaining 21.7% play once a month or less.^29^ Lastly, in a study focused on gaming centres, authors reported that among those who visit gaming centres, 95% do so for at least 1 hour per day.^27^

#### Prevalence of gaming

Six studies, all of which focused on young people, reported the prevalence of gaming in their samples (table 1). The prevalence of gaming ranged from approximately 20% to 78%.^25 26 29 33^ Two studies conducted in the same cohort in South Korea reported the prevalence of use of gaming centres to be approximately 60%.^27 28^

#### Study outcomes

Details related to study outcomes are shown in table 1. Five studies evaluated associations or correlations of gaming with self-reported hearing loss,^23^ audiometric thresholds,^27 31 33^ or tinnitus.^28^ Two of those studies presented associations after adjustment for relevant confounders,^27 28^ two presented unadjusted correlations or associations^31 33^ and the modelling approach was unclear in one study.^23^

Two studies conducted in South Korean middle and high school students within the same cohort found that after multivariable adjustment, use of gaming centres (vs non-use) was associated with increased odds of severe tinnitus and increased odds of bilateral high-frequency hearing loss but not speech-frequency hearing loss.^27 28^ Furthermore, one of those studies reported individuals who used gaming centres for a greater cumulative time were more likely to experience high-frequency hearing loss.^27^ Another large cross-sectional cohort study reported that playing egames was associated with increased odds of self-reported hearing loss severity.^23^ One study reported a correlation between the number of gaming centre visits per month and audiometric thresholds at 4.0 kHz in the right ear only, but did not report whether hearing thresholds at other frequencies were measured.^31^ That study did not detect correlations of hearing thresholds with frequency or duration of earphone use or duration of gaming centre visits.^31^ Finally, another study reported an association of gaming via headphones with aural fullness, but not with high-frequency or speech-frequency hearing loss.^33^

Three studies evaluated differences in gaming behaviours between males and females. Taken together, results indicated that boys (vs girls) played video games more often, for longer periods of time and at higher sound intensity levels.^25 26 30^ No studies evaluated whether associations of gaming with hearing loss and/or tinnitus differed by sex.

Next, a study conducted with data from nearly 37 000 participants of the household-based National Health Interview Survey (NHIS) extrapolated survey results on sound exposure from video or computer games (defined as at least 10 exposures per year) to the general USA population. Authors reported over 10 million individuals in the USA may be exposed to ‘loud’ or ‘very loud’ sound levels from video or computer games.^24^ Two studies evaluated use of hearing protection during activities with high-intensity sound levels, including gaming, and reported that use of hearing protection is uncommon during gaming.^23 24^

### Non-cohort observational studies

Three studies, the details for which are summarised in table 2, measured sound levels from video game consoles or gaming centres and did not take measurements on human subjects.^34^,^36^ One study measured the sound output of headphones attached to a gaming console to describe the typical sound levels experienced by users of five different video games.^34^ Two other studies aimed to evaluate sound levels in several gaming centres or arcades.^35 36^ Importantly, those two studies were conducted in years 1992 and 1983 and used arcade gaming systems as the source of video game audio, and thus may be limited in their comparability to modern gaming.^35 36^ All three studies described in this section additionally aimed to determine whether consistent exposure to these sound levels might exceed permissible levels of sound exposure.^34^,^36^

**Table 2:** Pertinent study characteristics from non-cohort observational studies that measured sound levels from actual video games or gaming centres.

First author (year)country	Purpose	Study design	Type and definition of gaming	Definition, sound level measure	Sound level of gaming	Primary study findings
Iannace *et al*^34^ (2020)Italy	Describe sound measurements in video game users in several scenarios produced by video games.	Observational	4 shooter video games and 1 racing game	Sound output of headphones attached to gaming console, reported as L_Aeq_ dB(A), L_Ceq_ dB(C), L_Cpeak_ dB(C)	L_Aeq_ range 84.6–91.2 dB(A) per game.L_Ceq_ range 88.7–93.0 dB(C) per game.L_Cpeak_ range from 104.9 to 118.7 dB(C) per game.	The sound levels of actual video games are high, and shooter games include several impulse noise events.
Mirbod *et al*^35^(1992)*Japan	Evaluate sound levels in recreational gaming centres including electronic arcade games.	Observational	Gaming centre	Sound survey, 192 samples were taken over 16 min in three gaming centres with sound level metre.Reported as L_Aeq_ and L_Ceq_.	Mean LAeq range from 90.2 to 92.0 dB(A) across three gaming centres.	The sound levels of games in gaming centre during actual operating conditions are high.
Plakke^36^ (1983)*USA	Measure sound levels of electronic games in arcades at normal and maximum operating levels.	Observational	Gaming centre	Sound survey conducted in 2 gaming centres with dosimeter.Measurements taken when games were set to their normal and maximum volumes.Reported as L_Aeq_.	Normal settings: range from 73 to 111 dB(A).Maximum settings range from 84 to 111 dB(A).	Most games had normal settings above 80 dB(A). The sound levels of gaming centres during operating conditions are high and may exceed permissible levels.

* Studies were published over 20 years ago and may be limited in their comparability to modern gaming.

dB, decibel; dB(A), decibel, A weighted; dB(C), decibel, C weighted; L_Aeq_, equivalent continuous sound level, A weighted; L_Ceq_equivalent continuous sound level, C weightedL_Cpeak_equivalent peak sound level, C weighted

Studies presented results in terms of A- or C-weighted average sound levels (L_Aeq_, L_Ceq_) and C-weighted peak sound pressure levels (L_Cpeak_). In the study that measured sound levels of five video games (recorded in a setting with low background noise) through headphones attached to the gaming console, the average sound levels were 88.5, 87.6, 85.6 and 91.2 dB(A) for four separate shooter games, and 85.6 dB(A) for a racing game.^34^ Authors reported peak sound pressure levels (L_Cpeak_) as 117.3, 118.7, 116.5, and 113.4 dB(C) for the shooter games and 104.9 dB(C) for the racing game.^34^ Finally, authors used average sound level values combined with periods of quiet to calculate the daily sound exposure levels for the following situations: 1 hour of play (7 hours of quiet), 2 hours of play (6 hours of quiet), 4 hours of play (4 hours of quiet) and 8 hours of play (0 hours of quiet). For example, with 4 hours of game play, the estimated sound exposure level across the five games is as follows: shooter games: 79.0, 78.6, 75.9, 81.5 dB(A), racing game: 78.1 dB(A). With 8 hours of game play, the sound exposure levels increase to: shooter games: 82.0, 81.6, 78.9, 84.5 dB(A), racing game: 81.1 dB(A). Authors conclude that the daily level of sound exposure from video games is close to maximum permissible levels of sound exposure.^34^

The next two studies were conducted in gaming centres in years 1992 and 1983, and therefore may not be representative of sound exposure in modern settings.^35 36^ One study measured sound levels, using a sound level metre, in three separate gaming centres with electronic arcade games, while games were being operated during actual operating conditions.^35^ Each gaming centre had between 18 and 28 games, and 12 to 14 sound samples were taken to estimate mean sound levels. The mean sound pressure levels were 90.2, 91.5 and 92.0 dB(A).^35^

The third study measured sound levels in two gaming centres with electronic arcade games, when the volume was set to (1) normal settings, and (2) maximum settings.^36^ Dosimeter microphones were placed within 3 inches of players’ ears to estimate the sound level reaching individual gamers, and therefore, sound measurements also captured the ambient noise in the arcade. In the first arcade, normal and maximum sound levels from seven games and two games, respectively, were measured, and in the second arcade, normal and maximum sound levels from 11 and two games, respectively, were measured. Two measurements are presented for each game. In the first arcade, average sound levels ranged from 73 to 93 dB(A) with normal settings. Of 14 sound measurements, two were between 73 and 75 dB(A), eight were between 82 and 85 dB(A) and four were between 92 and 93 dB(A). When set to maximum volume, sound levels were 99 to 101 dB(A) for one game, and 84 to 85 dB(A) for the other. In the second arcade, sound levels ranged from 83 to 111 dB(A) with normal settings. Of the 22 measurements made, 18 were between 83 and 89 dB(A), 2 were 91 and 92 dB(A) and 2 were 109 and 111 dB(A). When set to maximum volume, sound levels were 91 to 92 dB(A) for one game and 109 to 111 dB(A) for the other.^36^

### Studies that mention gaming and grey literature sources

An additional 16 peer-reviewed articles and 14 grey literature sources (four abstracts or conference papers, four newsletters or magazines, one letter to editor, five theses or dissertations) mention gaming as a potential source of excessive sound exposure, but none cite empirical evidence showing an association between gaming and hearing loss.^937^,^65^

One additional grey literature source (dissertation) aimed to evaluate (1) the preferred listening levels of individuals while playing a video game through headphones, and (2) whether there were changes to audiometric thresholds (0.25, 0.5, 1.0, 2.0, 3.0, 4.0, 6.0, 8.0 and 12.5 kHz) or distortion product otoacoustic emissions (1.0, 1.4, 2.0, 2.8, 4.0, 6.0 and 8.0 kHz) immediately after video game play, as compared with measurements taken before game play.^66^ In this experiment, 30 individuals with normal hearing played a shooter game for 20 min and used headphones set to their preferred listening levels. Results indicated that (1) preferred listening levels ranged from approximately 60 to 90 dB sound pressure level, and (2) there were significant increases (range 1–2 dB hearing level) to audiometric thresholds at 2.0, 6.0, 8.0 and 12.5 kHz (averaged across both ears) but not to distortion product otoacoustic emissions after game play. Authors conclude that gaming headphones can reach unsafe listening levels, which could place some gamers at risk of sound-induced hearing loss.^66^

## Discussion

The limited available evidence collated in this systematic scoping review indicates that gaming may be a source of unsafe listening. Therefore, gamers who are listening at high-intensity sound levels and for long periods of time may be at risk of permanent sound-induced hearing loss and/or tinnitus. Findings from this study highlight the need for additional research focused on the risk of hearing loss and/or tinnitus from gaming. Furthermore, findings suggest that there may be a need to prioritise interventions, such as initiatives focused on education and awareness of the potential risks of gaming, that can help promote safe listening among gamers.

Only two studies published in the last 10 years objectively measured average sound levels from video games or at gaming centres and reported high sound levels at approximately 84.6 to 91.2 dB(A) and 87.7 dB(A), respectively.^31 34^ Furthermore, three studies reported that individuals spend an average of 3 hours per week playing video games.^25 26 31^ Based on WHO permissible noise exposure levels described earlier (exposure level 75 dB, 40 hours per week, 3 dB exchange rate), the maximum number of hours per week a young person can safely listen at 83 dB, 86 dB and 92 dB is 6.5 hours, 3.25 hours and 45 min, respectively. For adults (exposure level 80 dB, 40 hours per week, 3 dB exchange rate) the maximum hours a week an individual can safely listen at those sound levels increases to 20 hours, 10 hours and 2.5 hours, respectively. As mentioned earlier, impulse sounds are also common in gaming and the presence of impulse noise is not necessarily captured by average sound intensity levels. One study reported that impulse sounds reached levels as high as 119 dB(C) during game play.^34^ This is important because impulse sounds may have additional negative impacts on hearing.^19^ Although the data provided in this review are limited, they suggest that some gamers, particularly those who play frequently, and at or above the average sound levels described by papers included in this review, probably exceed permissible sound exposure limits, and are thus engaging in unsafe listening practices, which could put them at risk for developing permanent hearing loss and/or tinnitus.^7^ Importantly, no studies in this review focused on esports, either for the individual esports gamer, or for esports spectators, as a potential source of unsafe listening, which highlights an important gap in the scientific literature. Given that esports are rapidly growing in popularity, research to understand the potential impacts of esports gaming on hearing, for both players and spectators, is warranted.

The prevalence of gaming and thus, the population who may be at risk of hearing loss and/or tinnitus from unsafe listening, probably varies by demographic factors, such as region, sex and age. An online source suggests that video game revenues are highest in Asia Pacific and North America, although video games are played worldwide, and that most gamers are under 18 (24%) or between 18 and 34 (36%) years of age.^5^ Along these lines, seven of the 14 studies in this review were conducted in Asia (South Korea, Indonesia, Japan, China), whereas the others were conducted in Australia, Europe and North America. While no studies in this review evaluated sex differences in associations between gaming and hearing or tinnitus, study results indicated that males play video games more frequently, for longer periods of time, and at higher-intensity sound levels.^25 26 30^ Of the cohort studies included in this review, all but one included children or young people in their samples. This is consistent with the source mentioned above that indicates the prevalence of gaming is probably highest in children and young adults. There may be other demographic factors not described in the studies included in this review, such as socioeconomic position, which might also be associated with unsafe listening practices among gamers.^67^ Future research is needed to identify populations at high risk of unsafe listening during gaming, which could inform targeted intervention strategies to prevent hearing loss and/or tinnitus among gamers.

Several studies included in this review reported associations or correlations of gaming with hearing loss and/or tinnitus, yet these relationships were detected only for certain outcomes such as high-frequency, but not speech-frequency, hearing thresholds, and one study did not report an association of gaming with hearing thresholds.^23 27 28 31 33^ Furthermore, some studies have reported inconsistent associations of other sources of leisure sound exposure, such as use of personal listening devices, with permanent changes to hearing.^68^,^71^ These inconsistent results may be due, in part, to differences in study methodology, such as study sample composition and/or definitions of gaming-related and hearing-related measures (eg, self-reported vs audiometric). It is likely that the type of gaming and the setting in which it occurs (ie, in gaming centres, on PCs or on mobile phones) influences these relationships. In this review, relationships of gaming with hearing loss or tinnitus were observed in studies that focused on gaming centres and PC gaming. However, most studies provided few details on the type of gaming and the setting in which it occurred, which highlights the need for future research to comprehensively evaluate these relationships. In addition to differences in study methodology, inconsistencies in research may be explained by the incremental and progressive nature of noise-induced hearing loss,^72^ which makes it difficult to capture short-term effects of sound or noise exposure, particularly in young people.

The WHO has recognised unsafe listening practices as an important global public health problem, given the negative consequences of sound or noise exposure on auditory and non-auditory health,^7^ and the fact that these consequences can be prevented through the practice of safe listening.^2 6^ The WHO ‘Make listening safe’ initiative, launched in 2015, aims to ensure that individuals of all ages can enjoy listening while protecting their hearing.^7^ A primary goal of ‘Make listening safe’ is to empower individuals to make choices that will regulate exposure to high-intensity sounds via education and provision of relevant resources.^2 3 6^ For example, materials available to the general public can be used to promote preventative strategies for individuals, and include recommendations such as limiting the time spent engaging in noisy activities, and keeping the volume at safe levels.

Along with the ‘Make listening safe’ resources available to individuals,^2^ there are global standards, including ‘Guidelines for safe listening devices/systems’ (ITU-T H.870) to describe requirements for safe listening on devices and systems, particularly those that play music.^15^ However, at this time, these guidelines do not apply to gaming consoles or devices. Importantly, to the authors’ knowledge, there are little to no global policy recommendations that focus on promoting safe listening among gamers. Results from this review suggest that policy development focused on safe listening among gamers at high risk for developing hearing loss and/or tinnitus, following the necessary supporting research, is warranted.

### Strengths and limitations

To the authors’ knowledge, this is the first article to comprehensively review whether gaming might be a source of unsafe listening. This information can be used to inform the need to prioritise research on this topic, and the development and implementation of policy to promote safe listening. This scoping review captures the strengths and limitations of the studies included in the review. For example, there were relatively few studies that evaluated associations of gaming and hearing loss and/or tinnitus, and those studies used varying methods, which makes it difficult to compare findings across studies. Given the nature of this scoping review, we did not assess risk of bias of the included studies. A single reviewer performed the title/abstract screen, which could introduce error into the process for determining study inclusion and exclusion. Although we conducted the systematic literature search in English, Spanish, and Chinese, there may be published articles in other languages that were not captured.

## Conclusions

The limited available evidence suggests that gaming may be a common source of unsafe listening, which could place many individuals worldwide at risk of hearing loss and/or tinnitus. Results suggest that additional research on these relationships is needed, including identifying populations of gamers at high risk of unsafe listening. These would help to inform and refine interventions that can prevent hearing loss and/or tinnitus among gamers. An important intervention could be the development and implementation of policy to promote safe listening among gamers.

## supplementary material

10.1136/bmjph-2023-000253 online supplemental file 1

## Data Availability

Data sharing not applicable as no datasets generated and/or analysed for this study.
