# Supplementary material for: Risk of sound-induced hearing loss from exposure to video gaming or esports: a systematic scoping review
Source: BMJ Public Health. 2024 Jan 4;2(1):e000253. doi: 10.1136/bmjph-2023-000253 (PMC11816424; doi:10.1136/bmjph-2023-000253)
Supplement: online supplemental file 1 [file bmjph-2-1-s001.pdf]

Online Supplemental File 1: Search strings for each database and grey literature searches. Searches were conducted January 17, 2023.

<u>Databases</u>

1. PubMed

Advanced search

n= 23

Filters: English

("video game" OR gaming OR esport*) AND ("noise-induced" OR "noise expos*" OR loud* OR “sound level” OR volume) AND (hearing OR tinnitus)

2. Web of Science core collection

Advanced search

n= 52

Filters: English

("video game" OR gaming OR esport*) AND ("noise-induced" OR "noise expos*" OR loud* OR “sound level” OR volume) AND (hearing OR tinnitus)

3. Scopus

Advanced search

n= 15

Filters: English

TITLE-ABS-KEY (("video game" OR gaming OR esport*) AND ("noise-induced" OR "noise expos*" OR loud* OR “sound level” OR volume) AND (hearing OR tinnitus))

Grey literature

Grey literature sources included white papers, newsletters, reports, proceedings, dissertations, theses, or published abstracts or conference papers. Sources were identified using the keywords (or derivations of) “hearing loss,” “noise induced,” and “video games” or “esports” using Google Scholar and other sources.
